# Development of a Nutrition Environment Assessment Tool for Latino Ethnic Stores

**DOI:** 10.3390/ijerph19031860

**Published:** 2022-02-07

**Authors:** Jenny L. Baier, Shelly M. Palmer, Donna M. Winham, Mack C. Shelley

**Affiliations:** 1 Mercy Hospital, Joplin, MO 64804, USA; jny.baier@gmail.com; 2 Department of Food Science & Human Nutrition, University of Illinois at Urbana-Champaign, Champaign, IL 61801, USA; s-palmer94@live.com; 3 Department of Food Science & Human Nutrition, Iowa State University, Ames, IA 50010, USA; 4 Departments of Political Science and Statistics, Iowa State University, Ames, IA 50010, USA; mshelley@iastate.edu

**Keywords:** food environment, Hispanic, NEMS-S, reliability, culture, food desert, food retail, dietary guidelines, ethnic market, Mexican American

## Abstract

The objectives were to: (1) adapt the Nutrition Environment Measures Survey for Stores (NEMS-S) to better culturally fit small Latino grocery stores (*tiendas*) in Iowa; (2) assess the newly adapted Latino NEMS-S for inter-rater and test-retest reliability; and (3) compare Latino and original NEMS-S summary scores. This pilot instrument, containing culturally appropriate foods from the original NEMS-S and 2015 US Dietary Guidelines for Americans, underwent two rounds of formative evaluation. The new instrument and scoring protocol were applied to a random sample of 42 of 81 possible *tiendas* in Iowa. Cohen’s kappa was used to assess inter-rater and test-retest reliability for availability and quality of indicator food items (total scores and food category sub scores). There were no differences in summary scores for inter-rater or test-retest reliability using paired *t*-tests. Inter-rater agreement was high (range 0.82–1.00; *p* < 0.001). *Tiendas* averaged 42.0 ± 7.5 of 57 possible points on the Latino NEMS-S, but only 12.0 ± 4.6 of 54 points on the original NEMS-S (*p* < 0.001). The Latino NEMS-S is a reliable tool for assessing the food environment within Iowa *tiendas*. Culturally specific instruments can describe diverse food environments more accurately and guide public health nutrition interventions within communities.

## 1. Introduction

Access to healthy foods has a primary role in shaping individuals’ diets, which can positively affect life-long health outcomes. The term ‘nutrition environment’ describes the characteristics of food outlets and food items available to consumers in a community [1]. While it is only one element of the complex network governing health and food choices, the nutrition environment enables or restricts eating behaviors. A lack of healthy, quality, and affordable foods accessible to consumers can contribute to chronic disease risk [1]. If healthy, affordable foods of suitable quality are not readily available in grocery stores, then shoppers, regardless of their intentions, are unable to obtain them. In fact, greater fruit and vegetable availability in stores may increase the likelihood of purchase [2].

The Nutrition Environment Measures Survey for Stores (NEMS-S) was developed to estimate characteristics of food items within retail grocery stores [1,3]. It is an observational tool used to assess availability, quality, and pricing for 11 food types (fruits, vegetables, milk, ground beef, hot dogs, frozen dinners, beverages, baked goods, bread, chips, and cereal) [1]. Except for fruits and vegetables, items were chosen based on common foods that contribute the highest amount of fat and calories in the American diet and their healthier alternatives [1,4,5]. Stores received higher NEMS-S scores if healthier versions of the high fat, high calorie foods are present (e.g., milk), if the healthier foods are lower or equal to the price of the less healthy items, and if fresh produce is of ‘acceptable quality’ (defined as peak condition, good color, fresh, firm, and clean) [1].

While the NEMS-S is the most widely used nutrition environment evaluation tool, it may not capture the diversity of healthy foods eaten by all US population groups due to the measurement options provided on the instrument [3]. The NEMS-S does not reflect foods commonly eaten by other ethnic groups such as Hispanics, nor does it consider food items that may be less healthy beyond dietary fat and calories, (e.g., added salt, added sugars, type of fat). Thus, the NEMS-S may only partially describe the nutrition environment of ethnic enclaves. Food deserts, usually found in low-income areas, are classified as zones without access to supermarkets, supercenters, or large grocery stores [6]. Accessibility to quality healthy foods that meet dietary needs and preferences can be a challenge, especially for those living in rural areas that may also be food deserts [7]. Unfortunately, ethnic markets or other stores unauthorized to accept Supplemental Nutrition Assistance Program (SNAP) benefits are not considered in the evaluation criteria for the purpose of defining food deserts [6].

Hispanics, defined as people with origins from Spanish-speaking countries, comprise 18.7% of the US population, and are the largest minority group at 62.1 million people [8]. ‘Latinos’ are people who have ancestral origins from Latin America, including Brazilians and Haitians whose native language is not Spanish. Most US Hispanics are of Latin American origin (Mexican 61.4%, Central American 9.8%, Puerto Rican 9.6%) [9]. US federal agencies report data using the ‘Hispanic’ classification. Although the two definitions overlap, this manuscript uses Latino, or the original source term as appropriate.

As of July 2019, Latinos comprised about 6.3% of the population in Iowa, or approximately 198,550 individuals. Of these, 74.8% were of Mexican ancestry and 29.7% were foreign-born. Latinos are projected to be 12.1% of Iowa’s population by 2050 [10]. Not all Hispanics identify with their ancestral cultures, but for many with Latin American roots, traditional foods are central to family and social life. Latino foods, such as beans, corn tortillas, lean meats, and fresh fruits and vegetables, are considered healthy and recommended by the 2015–2020 Dietary Guidelines for Americans (DGA) [11]. The inclusion of these foods in an adapted NEMS-S may more accurately describe Latino markets and food environments [12,13,14,15].

Food habits are one of the last characteristics to change during acculturation and often persist generations after immigration [12,13]. Retention of positive dietary habits depends on food availability. Dietary acculturation, the change in food habits to those of the host country, can lead to a decline in fruit, vegetable, and pulse (dry beans, dry peas, lentils, chickpeas) consumption and an increase in higher-fat convenience foods, sugar-sweetened beverages, and fast foods [12,13,14,15]. Hispanics in the US historically have had a higher prevalence of type 2 diabetes (14.0%) in comparison with non-Hispanic Whites (NHW) (6.0%) [16]. Obesity rates are also higher among Hispanics than for NHW (39.9% vs. 32.4%) [16]. More Hispanics met fruit (15.7%) and vegetable (10.5%) intake recommendations than NHW (12.2% and 9.3%, respectively) [17]. For pulses, 77% of Hispanics reported consuming them at least once per week in contrast to 63% of NHW and 52% of non-Hispanic Blacks [18].

*Tiendas* (i.e., small Latino grocery stores) and their culturally relevant food products within communities reinforce social acceptability and preservation of cultural traditions [19]. In addition, *tiendas* serve a social purpose by providing familiar foods, language, and community interaction for Latino cultures [19,20]. Thus, documentation of food availability at *tiendas*, due to their influence, would greatly contribute to knowledge of the nutritional environment for Latino populations. However, there is little information about the specific food options available in Latino food stores [13,19,20,21]. *Tiendas* may be the only accessible market in some locations and a preferred shopping venue for less acculturated Latinos. Many *tiendas* are independently owned, do not have electronic tracking of sales, and order from specialty grocers in larger cities. Unlike with mainstream supermarket chains, there are few ways to track food content or availability [19,21].

Prior to the current study, several NEMS-S adaptations for Latino neighborhoods or nutrition environments were created in the US. Emond et al. examined differences in Latino and non-Latino grocery stores to compare prices but used a different audit tool than the NEMS-S [21]. The Texas Nutrition Environment Assessment for Stores (TxNEA-S) modified the original NEMS-S to incorporate foods specific to Texas, including its Latino heritage, based on input from an expert panel [22]. For example, the presence of mangoes, tortillas, rice, beans, and other legumes were evaluated at grocery stores and convenience markets, but not specifically at Latino *tiendas*. TxNEA-S is unique in assessing canned and frozen produce, and beans [22]. A Guatemalan NEMS-S adaptation was compared to the NEMS-S for healthy food evaluation among Latino stores in Boston Massachusetts [23]. Healthy food availability within Latino stores was assessed with the addition of Guatemalan specific fruits, vegetables, canned beans, and ultra-high temperature pasteurized (UHT) boxed milk in the Guatemalan NEMS-S, items not present in the original instrument [23]. These other adaptations mainly focused on culturally specific fruits and vegetables. Despite these advancements, instruments that go beyond fresh produce and include a range of healthy cultural foods in other categories are still needed [3]. We developed and evaluated a comprehensive nutrition environment assessment suitable for Latino *tiendas* in Iowa. This instrument can provide data needed to formulate public health outreach interventions with this vulnerable emerging population [10,19,20].

The objectives of the study were to: (1) adapt the NEMS-S for use in *tiendas* in Iowa; (2) quantify the inter-rater and test-retest reliability of the resulting Latino NEMS-S; and (3) compare scores of the Latino NEMS-S to the original NEMS-S to document differences in healthy food assessment by tool choice.

## 2. Materials and Methods

### 2.1. Instrument Origins

The Latino NEMS-S represents an evolution and revision of the Mexican/Mexican-American NEMS-S (NEMS-S Mex) instrument protocol developed by Szkupinski Quiroga and the principal investigator (PI; D.M.W.) [24]. The NEMS-S Mex was pilot tested in metropolitan Phoenix, Arizona, with 32 Latino stores ranging from large supermarkets to medium and small *tiendas* [24]. Diversity in the store sample resulted in too few stores similar enough in size and store type for adequate inter-rater and test-retest rater reliability comparisons. The PI was invited to collaborate with the Iowa Department of Public Health “Shop Healthy Iowa” program. This intervention provided small Hispanic retail stores with promotional materials and product placement guidance to increase sales of fruit and vegetable varieties [25]. “Shop Healthy Iowa” needed a suitable nutrition environment measure to track pre- and post-intervention changes in stores. The researchers planned to test the NEMS-S Mex instrument with a larger sample of Latino stores in Central Iowa [24]. To ensure no major changes had occurred that would require adjustment of the NEM-S Mex food items, the researchers reviewed the 2009–2012 National Health and Nutrition Examination Survey (NHANES) frequency consumptions for Hispanics, and examined Latino food market trends [12,19,20,26]. No changes in national consumption patterns were observed from the NEMS-S Mex. Between February and June 2015, 22 *tiendas* in Central Iowa were evaluated with the NEMS-S Mex instrument to confirm if it would meet the needs of “Shop Healthy Iowa.”

After viewing the stores and analyzing these pilot data, the formative evaluation revealed that the Latino store structure, items stocked, and food quality were markedly different in Iowa compared to Arizona. With fewer food items, less variety, and smaller size, neither the original NEMS-S nor the NEMS-S Mex were suitable for the Iowa *tiendas*.

### 2.2. Development of Latino NEMS-S Iowa

To tailor and update a new Latino store instrument to Iowa, the NEM-S Mex data from the 22 *tiendas* were examined in light of the 2015–2020 DGA’s MyPlate healthy food group guidance regarding fruits, vegetables, grains, protein foods, and dairy [11,27]. The 2009–2012 NHANES Hispanic consumption data were used to inform food choices. Instrument content validity is thus supported by not only US national food group recommendations, but also national consumption data, and in-person store observations [1,26]. Categories for vegetables (green, orange, red, legumes, starchy), fruits, and fat-free or low-fat dairy were expanded on the draft instrument. Additional measures were added for whole grains (brown rice, whole wheat, and corn tortillas), varied protein sources (eggs, beans, beef, chicken, fish), solid fats and oils, reduced added sugars (light syrup for canned fruit, 100% juice), reduced saturated fats (chicken, lean beef), and reduced sodium intake (low-sodium canned vegetables and beans). The 2015–2020 DGA emphasized consumption of 100% juice and advocated for decreasing foods with added sugar [11]. Based on known high-consumption patterns of fruit nectars and canned fruits among US Latinos, fruit nectars and canned fruit packaged in light syrup and 100% juice were added to the Iowa instrument to evaluate availability of these products and healthier alternatives. DGA guidelines also highlighted the importance of limiting saturated fats to <10% of kilocalories per day and choosing lower-fat versions of foods (e.g., milk, meat cuts) [11]. The 2015–2020 DGA specifically recommended consuming foods rich in shortfall nutrients of concern, including dietary fiber, potassium, calcium, and vitamin D [11]. These nutrients are found in healthful foods on the Latino NEMS-S (Table 1). In a separate nutrition knowledge survey of Midwestern consumers, most Latino respondents identified the ‘healthier’ food options included on the Latino NEMS-S which further contributes to the face validity of the instrument [20].

### 2.3. Pilot Testing of Latino NEMS-S Instrument

Pilot testing of 24 *tiendas* using the preliminary version of the Latino NEMS-S was conducted to gain insight into the instrument’s efficacy. The original NEMS-S included referent food items within its categories. Documentation of the typical foods, brands, and item packaging available in *tiendas* was needed to guide standardization of referent food items on the Latino NEMS-S. Data were also collected using the original NEMS-S at these same stores. Cheeses, *pan dulce*, frozen fruits and vegetables, and organic foods (eggs, fruit, and vegetables), Latino culture equivalents for frozen dinners, bread, baked chips, and cereal were included. These were later dropped due to inconsistent availability during pilot testing (Table 2).

Beefsteak categories were rarely labeled and difficult to distinguish without asking the butcher. Interviews with store employees about available items were not routinely feasible during evaluations because most stores were staffed by only one worker. Due to these limitations, the survey was simplified to ask if lean (≤10%) and regular (>10% fat) beef were available rather than requiring raters to identify a specific cut as the referent item at all stores. Chicken was added to the final Latino NEMS-S as it is a frequently consumed source of lean protein by Hispanics according to NHANES [26]. The instrument compares the lean option of chicken breast with the regular higher fat version of legs and thighs. Eggs were added because they are a low-cost protein source with wide availability.

The final Latino NEMS-S contains 13 categories. These include dairy (milk, UHT milk), fruits (fresh and canned), vegetables (fresh and canned), grains (tortillas, rice), and protein (beefsteak, chicken, fish, eggs, and canned and dry beans—which were also considered vegetables). Beverages (water, fruit nectar) and fats (cooking oils, solid fats) were added. Table 2 shows the comparison of food categories within the original NEMS-S, the pilot Latino NEMS-S, and the final Latino NEMS-S for Iowa *tiendas*. Further details regarding the rationale for different foods tested in the Iowa formative surveys are available in Baier [28]. This information is useful for regional refinement of the instrument or other ethnic adaptations. The Latino NEMS-S instrument is available in the Supplementary Materials.

### 2.4. Identification, Selection, and Consent of Stores

Prior to formal evaluation of the Latino NEMS-S, a working list of known Latino food markets was developed based on field work, internet searches, telephone calls to prospective businesses, and the assistance of the Iowa State Extension & Outreach, Community and Economic Development office. The list of Latino food markets was verified and expanded by the research field team through direct observation and inquiry when in communities. A total of 81 Latino stores were located across Iowa. Most of these *tiendas* fit the classification of ‘corner stores’ defined as less than 2000 square feet, four aisles or less, and one cash register [29]. Several Latino stores were also home to a restaurant or other small business. The restaurant/small business was included in our evaluation if they had a separate cash register from the grocery store used within the restaurant/small business and if they met our other inclusion criteria.

**Figure 1:**
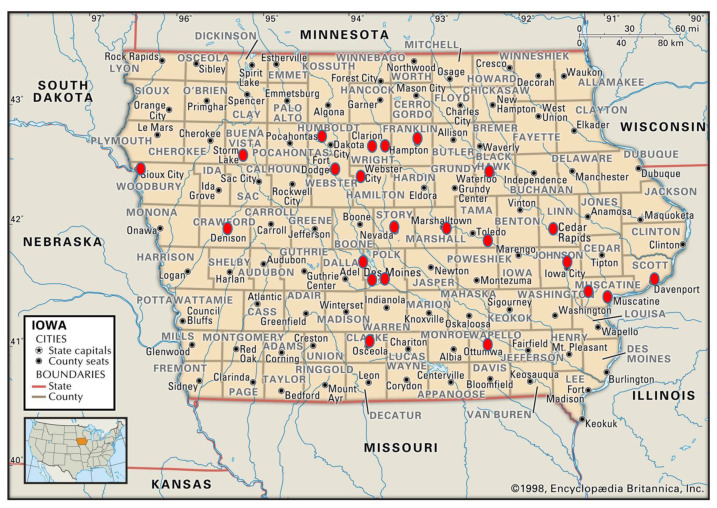
Location of *tiendas* in Iowa evaluated by the Latino NEMS-S.

Prior to data collection, all 81 eligible stores were mailed a letter of introduction in English and Spanish. The letter was intended to introduce the researchers and explain the study purpose. Store owners were asked to contact the researchers with questions or to state that they did not wish to participate. Two store owners called to ask for more information. No storeowners declined participation at that time. From the list of 81 stores, 47 were randomly selected for inter-rater reliability and test-retest reliability data collection using the random number function in Excel (Microsoft, Redmond, WA). Three *tiendas* in Southwest Iowa (Council Bluffs region) were subsequently excluded from sampling due to excessive geographic distance from other stores and researcher resource constraints. The geographic distribution of the sample is shown in Figure 1.

### 2.5. Latino NEMS-S Test Protocol

The eight-member research team trained on the original NEMS-S data collection protocol and methods prior to data collection as described in Section 2.2 and Section 2.3 [30]. Researchers (raters) completed the NEMS-S online training module and practiced evaluating three chain grocery stores. Questions and administration procedures were clarified for the original instrument during group debriefing and review sessions. Field training with the Latino NEMS-S was conducted at the same three local chain grocery stores used during rater practice and at the only large Latino supermarket in Iowa at the time. Inter-rater and test-retest reliability data were collected for the revised Latino NEMS-S from June to December 2016. Teams of two raters visited each store on two occasions approximately one week apart. Raters were randomized to their roles and stores by the PI in advance to reduce potential bias. Data collection proceeded as described below.

First Store Visit—Time 1 (T1): Inter-Rater Reliability. On the first visit (T1), a bilingual researcher described the study purpose to the store clerk or manager and provided a copy of the English-Spanish introduction letter. With permission, two raters independently completed the Latino NEMS-S. On exit, the team asked permission to return within two weeks to complete a second evaluation.

Second Store Visit—Time 2 (T2): Test-Retest. The T2 evaluation was completed within two weeks after T1. The same two raters visited each store for a second time. One of the two raters who had initially surveyed the store for T1 was randomly chosen to repeat the Latino NEMS-S. The second rater completed the original NEMS-S instrument. Mean instrument completion time was approximately 45 min for the Latino NEMS-S and 20 min for the original NEMS-S. Survey responses were subsequently entered into an Excel spreadsheet by a separate team member who was not at that specific store and entered a second time and cleaned by other research team members prior to analysis.

### 2.6. Development of Scoring System

A Latino NEMS-S scoring guide was modeled from the original NEMS-S to adjust for modified and added categories. The scoring system awarded points for availability of nutritious items and for fresh fruit and vegetable quality. Unlike the original NEMS-S scoring, no points were assigned based on food prices [1]. Most Iowa *tiendas* either did not label food prices or the listed price was inconsistent with the charged price [19]. The total maximum points possible is 57 for the Latino NEMS-S and 54 for the original NEM-S. Summary scores indicating overall food availability at each store were tallied and compared for each survey. The scoring guide is available in the Supplementary Materials.

### 2.7. Analysis of Data

Data entry and analyses were completed using SPSS (IBM, v. 26, Armonk, New York, NY, USA). Cohen’s kappa was used to determine inter-rater reliability between rater 1 and rater 2 with the Latino NEMS-S at each store for T1. This value was calculated for each of the food item variable measures to determine differences between raters. Kappa scores were also used to determine if the same items were available between T1 and T2 as a measure of test-retest reliability. Paired t-tests were completed assessing variation in scores between the Latino NEMS-S and original NEMS-S for each store.

## 3. Results

### 3.1. Characteristics of Sample Latino Grocery Stores

Of the 44 eligible Latino stores randomly selected for data collection, two were subsequently excluded from analysis. One store was not open on the second visit, and a second declined further participation. Figure 1 illustrates the geographic location of the 42 surveyed stores. The highest percentage of *tiendas* were in Northwest Iowa (31%), followed by the Southeast (29%), Central (21%), and Northeast (19%) regions. Eighty-three percent of the stores had a meat counter and 76% had a frozen food section. While 69% sold alcoholic beverages, only 12% sold tobacco products. Almost half of the *tiendas* sold hot made-to-order foods, and one had an attached Mexican restaurant. Eighty-one percent (*n* = 34) of the stores were in an area classified as low-income and with low-access to healthy foods, or food desert, by the United States Department of Agriculture’s (USDA) Food Access Research Atlas. Three of the stores were located in close proximity to a food desert area, and five were in rural areas not classified by the USDA because of low population density [6].

### 3.2. Scoring Results

Mean summary scores during T1 were 41.8 ± 7.6 for rater 1 and 41.6 ± 7.6 for rater 2 (correlation coefficient = 0.962). The test-retest summary scores were similar for T1 (41.8 ± 7.6) and T2 (41.9 ± 7.5) (correlation coefficient = 0.951). No significant differences were observed by paired *t*-tests for the inter-rater or test-retest overall scores.

**Figure 2:**
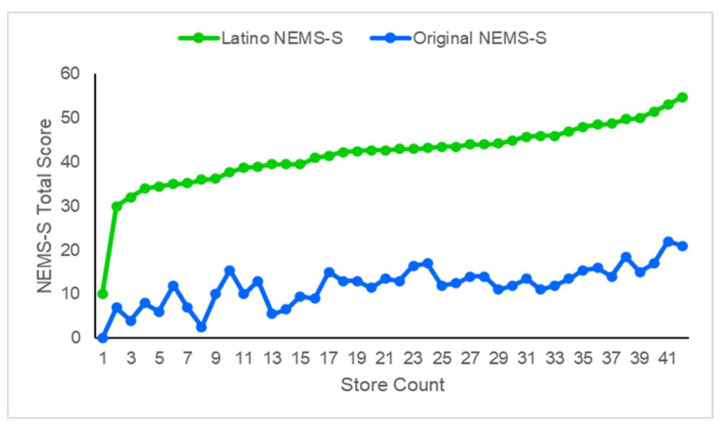
Latino NEMS-S and original NEMS-S total scores for each *tienda* (*n* = 42).

The average T2 score for the Latino NEMS-S was 41.9 ± 7.5, while the original NEMS-S was 12.0 ± 4.6. Latino NEMS-S scores ranged from 10 to 55 points, and original NEMS-S scores varied from 0 to 18.5 points. Higher scores on both instruments indicate greater availability of healthful food choices as compared to regular options, better availability and quality of fresh fruits and vegetables, as well as equal or lower price for the healthier option in the original NEMS-S. Figure 2 displays the Latino NEMS-S and original NEMS-S scores for each of the 42 *tiendas* evaluated.

### 3.3. Reliability

Table 3 includes kappa values for the individual healthy food options available. Inter-rater reliability values ranged from 0.90 to 1.00, and test-retest values ranged from 0.90 to 1.00 (average *p* value < 0.001).

Table 4 shows inter-rater reliability and test-retest kappa scores for fresh fruits and vegetables. Values ranged from 0.90 to 1.00 for inter-rater reliability and 0.80 to 1.00 for test-retest (average *p* value < 0.001). Agreement for raters was high, as was consistency in identification of products available between T1 and T2. These results indicate the Latino NEMS-S is reliable in assessing the food environment within Iowa *tiendas*.

## 4. Discussion

The main purpose of this study was to develop and test the reliability of a Latino NEMS-S instrument to determine food availability and accessibility in Iowa *tiendas*. As expected, when comparing scores between the Latino NEMS-S and original NEMS-S instruments, *tiendas* scored much higher with the culturally adapted instrument. This study fills an important research gap by providing a tool that more accurately describes food options in Latino *tiendas*. Prior to this instrument’s development, areas where Latino grocery stores were located may have been considered food deserts due to a lack of information about the culturally specific food items present.

Inter-rater and test-retest reliability on the Latino NEMS-S was high with Cohen’s kappa scores of almost perfect agreement [31]. These results are consistent with the original NEMS-S when the instrument was developed and tested in 85 stores [1]. High reliability measures with multiple raters suggest that consistent results will also be obtained by users with limited previous knowledge of the instrument.

Score consistency among raters may be attributed to a variety of factors. These include the training received in the survey directions, gaining familiarity with different packaging of foods and fruits and vegetables seen in the *tiendas*, and perhaps issues with the survey itself. While detailed, the survey is straightforward in describing what products to look for during the rating process and in differentiating which product receives precedence over another if variations are present.

In comparison to other ethnic store adaptations, three iterations of the survey were tested and analyzed to identify areas needing improvement. Like the original NEMS-S, food selections included in the instrument were drawn from national Hispanic food consumption data, based on the DGA, and availability of products in *tiendas*. These characteristics support the content validity of the Latino NEMS-S [1,3]. A separate survey with Midwest Latinos found high recognition of the healthy vs. less healthy food options which also supports the instrument’s face validity [20].

A large number of *tiendas* were surveyed in both urban and rural areas across the state. Thus, the Latino NEMS-S has been broadly tested in a variety of conditions and locations. Not only was there an increase in the amount of healthy foods documented in comparison to the original NEMS-S, food availability within the stores included all five food groups from MyPlate and would allow customers to meet 2015–2020 DGA nutrition recommendations [11,27]. As healthy foods can and do exist at *tiendas* in some of these supposed food deserts, our findings show that high-risk (low access/low income) people can find desired foods. In turn, these markets can promote retention of positive dietary habits [18,19]. Furthermore, many counties with Latino stores have high levels of poverty (Crawford 15.8%, Johnston 17.8%; Black Hawk 15.8%) [32]. The *tienda* can serve as a food environment resource for all members in a community beyond Latinos [19,20,21].

Given the findings of this study, it stands to reason that other researchers interested in creating a new modification of the original NEMS could model aspects of the survey and testing procedures completed during development of the Latino NEMS-S. There are numerous varieties of ethnic food stores serving the growing diverse US population that could benefit from such an analysis. A survey could measure healthy food options available, and once this is determined, research groups could go a step further by implementing strategies to educate and partner with store owners if there are any gaps present in the availability of healthy cultural foods [25].

### Study Limitations

Despite its strengths, the Latino NEMS-S instrument is not without limitations. The survey may favor foods from a Mexican-ancestry Latino subculture or region more than others. Seasonality and the time of year when the raters visited each *tienda* may also be a factor in assessing fruit and vegetable availability at a given timepoint for each store. Additionally, we cannot extrapolate findings for the Latino NEMS-S to other geographic areas outside of Iowa. Hence, the need for the variations of the NEMS-S to accommodate for the diversity of store types and the populations they serve.

Another study limitation comes from our inability to obtain one of the most important aspects in retail: the price. As previously noted, pricing at *tiendas* was not measured due to inconsistent usage of signage in the *tiendas*. Had this been available, pricing information would have provided researchers the ability to compare healthy and less healthy items, different Latino stores, geographical areas, and Latino stores versus mainstream grocery retailers.

## 5. Conclusions

An opportunity for creating sustainable nutrition changes in a community may be through targeting the nutrition environment [29,33]. This study provides examples of evaluating ethnic stores through direct comparison of food availability based on what is considered healthy by the US Dietary Guidelines. Culturally accurate assessments like the Latino NEMS-S can better inform relationships between the nutrition environment and healthy eating habits among vulnerable populations. *Tiendas* have the ability to provide access to high quality, affordable foods for all residents in underserved communities, not just Latinos. Recognition of their substantial contributions to food access in purported food desert regions is essential in developing guidance for nutrition and reducing health disparities. Increased recognition of ethnic food stores in communities may encourage families to retain positive dietary habits along the acculturation spectrum [12,13,14,15].

## Figures and Tables

**Table 1:** Latino NEMS-S survey items and relevance to the Dietary Guidelines for Americans [11].

NEMS-S Latino Categories	Healthier Option	Regular Option	2015 Dietary Guidelines Healthy Eating Pattern
Milk + UHT ^1^ milk	Skim or 1%	Whole; 2%	Fat-free or low-fat dairy
Eggs	White eggs, brown eggs	None	Variety of protein foods
Fresh fruits	Bananas, apples, oranges, grapes, cantaloupe, watermelon, pears, peaches, strawberries, honeydew	None	Fruits, especially whole fruits
Latino fresh fruits	Papaya, mango, pineapple, guava, plums, grapefruit, other banana plantains	None	Fruits, especially whole fruits
Canned fruits	Water, light syrup, 100% juice Pineapple, guava, cherries, mango, fruit cocktail, peaches	Heavy syrup	Fruits, especially unsweetened or no-sugar-added versions
Fresh vegetables	Tomatoes, lettuce, carrots, cabbage, cucumber, sweet peppers, corn, celery, broccoli, cauliflower	None	Variety from all subgroups Dark green/red/orange broccoli, carrots, tomatoes
Latino fresh vegetables	Tomatillo, onion, chilies, white potato, squash, avocado, radishes, jicama, cactus pad, spinach, sweet potato, plantains	None	Variety from all subgroups Dark green/red/orange: spinach, tomatillos; Starchy: Potato, corn, jicama
Canned vegetables	Low sodium Corn, green peas, cactus, mixed vegetables, green beans	Regular packaging	Variety from all subgroups Dark green/red/orange: mixed vegetables, cactus, green beans; Starchy: corn, peas
Juice + Juice nectars + water	Water, 100% juice, Healthy: Fruit Nectars Alternate: Mineral Water	Coca Cola Alternate: Sugared soda	Limit added sugar consumption to <10% of kcals
Beefsteak	Sirloin Tip Alternate: top round, flank steak, lean ground beef (≤10% fat)	Bottom Round Alternate: short ribs, regular ground beef (>10% fat)	Variety of protein foods
Chicken	Fresh chicken breast or white meat Alternate: frozen	Fresh legs and thighs (dark meat) Alternate: frozen	Variety of protein foods
Fresh and frozen fish	Fresh tilapia, cod, salmon, catfish, mojarra, other fish Alternate: frozen	None	Variety of protein foods
Canned fish	Sardines in tomato sauce; tuna and sardines in oil; tuna and sardines in water	None	Variety of protein foods
Tortillas	Healthiest: yellow and white corn Healthy:100% whole wheat or whole grain	Flour tortillas	Grains, at least half of which are whole grains
Canned beans	Healthiest: Low sodium whole canned beans Healthy: Whole canned beans	Refried beans	Variety of vegetable and protein foods: Legumes
Dry beans	Pinto, black, mayocoba, red	None	Variety of vegetable and protein foods: Legumes
Rice	Brown rice, white parboiled rice	White long grain rice; Alternate: Jasmine rice	Grains, at least half of which are whole grains
Cooking oils	Extra virgin olive oil, olive oil, canola oil, vegetable oil, corn oil	Lard	Oils; limit saturated (<10% calories) and trans fats

^1^ UHT = ultra-high temperature pasteurized milk.

**Table 2:** Original NEMS-S food categories compared to those of the Latino NEMS-S adaption.

Original NEMS-S	Pilot Latino NEMS-S (*n* = 24)	Final Latino NEMS-S (*n* = 42)
Milk	Milk + UHT ^1^ Milk	Milk + UHT Milk
-	Eggs	Eggs
Fruits	Fresh fruits	Fresh fruits
-	Latino fresh fruits	Latino fresh fruits
-	Canned fruits	Canned fruits
-	Frozen fruits	-
Vegetables	Fresh vegetables	Fresh vegetables
-	Latino fresh vegetables	Latino fresh vegetables
-	Canned vegetables	Canned vegetables
-	Frozen vegetables	-
Ground Beef	Beefsteak and ground beef	Beefsteak and ground beef
-	Fresh and frozen chicken	Fresh and frozen chicken
-	Fresh and frozen fish	Fresh and frozen fish
-	Canned fish	Canned fish
Hot dogs	Hot dogs	-
Frozen dinners	Frozen dinners + Latino frozen dinners	-
Baked goods	Baked goods + Mexican sweet bread	-
Beverages	Beverages + juice nectars + water	Beverages + juice nectars + water
Soda: Coca-Cola	Soda: Coca-Cola	Soda: Coca-Cola
Bread	Bread	-
-	Tortillas	Tortillas
Baked Chips	Chips	-
Cereal	Cereal	-
-	Canned beans	Canned beans
-	Dry beans	Dry beans
-	Rice	Rice
-	Cooking oils	Cooking oils
-	Solid fats	Solid fats

^1^ UHT = ultra-high temperature pasteurized milk.

**Table 3:** Inter-rater and test-retest reliability values for Latino NEMS-S dairy, meat, juice, and grains (*n* = 42).

	Inter-Rater Reliability		Test-Retest Reliability	
Type of Food	% Agreement	Kappa	% Agreement	Kappa
Milk	100.00	1.00	92.86	0.95
UHT milk ^†^	90.24	0.95	92.68	0.95
Eggs	97.62	0.98	78.57	0.85
Meat/Fish				
Lean beef	100.00	1.00	90.48	0.95
Chicken	100.00	1.00	90.48	0.93
Fresh fish	95.24	0.98	88.10	0.92
Frozen fish	95.24	0.98	88.10	0.92
Beverages				
100% juice	88.10	0.92	88.10	0.92
Nectar juice	92.86	0.96	95.24	0.98
Tortillas				
Corn tortillas	100.00	1.00	100.00	1.00
Whole wheat tortillas	95.24	0.97	90.48	0.95
Beans				
Low sodium canned beans	95.24	0.98	95.24	0.98
98–100% fat-free refried beans	95.24	0.97	95.24	0.98
Dry beans	100.00	1.00	100.00	1.00
Rice				
Brown rice	95.24	0.97	95.24	0.98
Parboiled rice	90.48	0.94	90.48	0.95
Canned fish	100	1.00	100	1.00

^†^ Mean of 41 stores. UHT = ultra-high temperature pasteurized milk.

**Table 4:** Inter-rater and test-retest reliability values for Latino NEMS-S fruit and vegetable assessment (*n* = 42).

	Inter-Rater Reliability		Test-Retest Reliability	
Type of Food	% Agreement	Kappa	% Agreement	Kappa
Fresh Fruit				
Bananas	90.48	0.94	78.57	0.86
Apples	100.00	1.00	90.48	0.94
Oranges	95.24	0.98	88.10	0.92
Grapes	95.24	0.98	92.86	0.96
Cantaloupe	95.24	0.98	85.71	0.90
Peaches	95.24	0.98	95.24	0.97
Honeydew melon	100.00	1.00	100.00	1.00
Watermelon	100.00	1.00	78.57	0.83
Pears	95.24	0.98	95.24	0.97
Plantains	90.48	0.95	95.24	0.97
Papaya	90.48	0.95	78.57	0.85
Mango	95.24	0.98	80.95	0.87
Pineapple	90.48	0.94	85.71	0.90
Guava ^y^	87.80	0.91	85.37	0.90
Grapefruit	95.24	0.98	95.24	0.97
Fresh Vegetables				
Tomatoes	100.00	1.00	100.00	1.00
Lettuce	85.71	0.90	88.10	0.92
Carrots	95.24	0.98	90.48	0.93
Cabbage	85.71	0.90	90.48	0.93
Cucumber	95.24	0.98	85.71	0.89
Sweet peppers	88.10	0.92	85.71	0.89
Corn	95.24	0.97	85.71	0.90
Celery	90.48	0.95	95.24	0.98
Tomatillos	95.24	0.97	100.00	1.00
Onion	100.00	1.00	95.24	0.98
Chiles	95.24	0.98	90.48	0.95
Potatoes	90.48	0.95	80.95	0.87
Squash	95.24	0.98	90.48	0.95
Avocado ^x^	90.00	0.92	72.50	0.82
Radishes	95.24	0.97	76.19	0.86
Jicama ^y^	92.68	0.95	82.93	0.90
Cactus pad	85.71	0.90	80.95	0.87
Sweet potato	95.24	0.97	90.48	0.93

^x^ Mean of 40 stores. ^y^ Mean of 41 stores.

## Data Availability

Data will be made available by request to the PI.

## Supplementary Materials

The following are available online at https://www.mdpi.com/article/10.3390/ijerph19031860/s1, S1: Latino NEMS-S instrument, S2: Latino NEMS-S protocol guide; S3: Latino NEMS-S Scoring Guide; S4: Latino NEMS-S Glossary of Foods.

## References

[1] Glanz K., Sallis J.F., Saelens B.E., Frank L.D. (2007). Nutrition environment measures survey in stores (NEMS-S): Development and evaluation. Am. J. Prev. Med..

[2] Martin K.S., Havens E., Boyle K.E., Matthews G., Schilling E.A., Harel O., Ferris A.M. (2012). If you stock it, will they buy it? Healthy food availability and customer purchasing behavior within corner stores in Hartford, CT, USA. Public Health Nutr..

[3] Glanz K., Johnson L., Yaroch A.L., Phillips M., Ayala G.X., Davis E.L. (2016). Measures of retail food store environments and sales: Review and implications for healthy eating initiatives. J. Nutr. Educ. Behav..

[4] Nielsen S., Popkin B. (2003). Patterns and trends in food portion sizes, 1977–1998. JAMA.

[5] U.S. Department of Health and Human Services, U.S. Department of Agriculture (2005). Dietary Guidelines for Americans.

[6] United States Department of Agriculture Food Access Research Atlas. https://www.ers.usda.gov/data-products/food-access-research-atlas/documentation/.

[7] Bell J., Mora G., Hagan E., Rubin V., Karpyn A. Access to Healthy Food and Why It Matters: A Review of the Research. The Food Trust: Philadelphia, PA, USA. http://thefoodtrust.org/uploads/media_items/access-to-healthy-food.original.pdf.

[8] Jones N., Marks R., Ramirez R., Rios-Vargas M. 2020 Census Illuminates Racial and Ethnic Composition of the Country. https://www.census.gov/library/stories/2021/08/improved-race-ethnicity-measures-reveal-united-states-population-much-more-multiracial.html.

[9] U.S. Department of Health and Human Services Office of Minority Health Profile: Hispanic/Latino Americans. https://minorityhealth.hhs.gov/omh/browse.aspx?lvl=3&lvlid=64.

[10] State Data Center of Iowa (2020). Latinos in Iowa. https://www.iowadatacenter.org/Publications/latinos2020.pdf.

[11] U.S. Department of Health and Human Services, U.S. Department of Agriculture (2015). 2015–2020 Dietary Guidelines for Americans. 8th ed. http://health.gov/dietaryguidelines/2015/.

[12] Kerber C., Kessler L., Wallace S., Burns-Whitmore B. (2014). Cultural and dietary factors influencing traditional Latino meal patterns: Findings from focus group discussion. Calif. J. Health Promot..

[13] Winham D.M. (2009). Culturally tailored foods and cardiovascular disease prevention. Am. J. Lifestyle Med..

[14] Ayala G.X., Baquero B., Klinger S. (2008). A systematic review of the relationship between acculturation and diet among Latinos in the United States: Implications for future research. J. Am. Diet. Assoc..

[15] Heer M.M., Winham D.M. (2020). Bean preferences vary by acculturation level among Latinas and by ethnicity with Non-Hispanic White women. Int. J. Environ. Res. Public Health.

[16] Dominguez K., Penman-Aguilar A., Chang M.H., Moonesinghe R., Castellanos T., Rodriguez-Lainz A. (2015). Centers for Disease Control and Prevention (CDC). Vital signs: Leading causes of death, prevalence of diseases and risk factors, and use of health services among Hispanics in the United States—2009–2013. MMWR Morb. Mortal. Wkly. Rep..

[17] Lee-Kwan S.H., Moore L.V., Blanck H.M., Harris D.M., Galuska D. (2017). Disparities in state-specific adult fruit and vegetable consumption—United States, 2015. MMWR Morb. Mortal. Wkly. Rep..

[18] Tichenor N., Conrad Z. (2016). Inter-and independent effects of region and race/ethnicity on variety of fruit and vegetable consumption in the USA: 2011 Behavioral Risk Factor Surveillance System (BRFSS). Public Health Nutr..

[19] Bates L. (2017). Latino groceries in the rural Midwest: An examination of food security, cultural identity, and economics. Leopold Cent. Complet. Grant Rep..

[20] Palmer S.M., Winham D.M. (2020). Midwest consumer shopping habits, nutrition knowledge, and Latino *tienda* use. Health Behav. Policy Rev..

[21] Emond J., Madanat H., Ayala G. (2012). Do Latino and non-Latino grocery stores differ in the availability and affordability of healthy food items in a low-income, metropolitan region?. Public Health Nutr..

[22] Gloria C., Steinhardt M. (2010). Texas nutrition environment assessment of retail food stores (TxNEA-S): Development and evaluation. Public Health Nutr..

[23] Caplan E., Kanter R., Bearup R., Solomons N.W., Bermudez O.I. (2017). Comparative performance of NEMS-S Surveys in Latino food stores in the greater Boston area. Arch. Latinoam. Nutr..

[24] Winham D.M., Szkupinski Quiroga S. (2013). Adaptation of the Nutrition Environment Measures Survey-Stores (NEMS-S) to assess a Mexican/Mexican-American nutrition environment. FASEB J..

[25] Compre Saludable/Shop Health Iowa. https://idph.iowa.gov/inn/compre-saludable.

[26] Food Surveys Research Group, U.S. Department of Agriculture, Agricultural Research Service Nutrient Intakes from Food and Beverages: Mean Amounts Consumed per Individual, by Gender and Age, What We Eat in America, NHANES 2001–2012. http://www.ars.usda.gov/Services/docs.htm?docid=18349.

[27] United States Department of Agriculture Choose MyPlate. https://www.choosemyplate.gov/.

[28] Baier J.L. (2017). Development of Tool to Measure the Latino Market an Assessment Nutrition Environment. Master’s Thesis.

[29] The Food Trust Philadelphia’s Healthy Corner Store Initiative: 2010–2012. http://thefoodtrust.org/uploads/media_items/hcsi-y2report-final.original.pdf.

[30] Nutritional Environment Measures Survey. https://nems-upenn.org/.

[31] Viera A.J., Garrett J.M. (2005). Understanding interobserver agreement: The kappa statistic. Fam. Med..

[32] Iowa State Data Center Iowa County Poverty Rates. https://www.iowadatacenter.org/data/acs/econ/poverty/couty-poverty-map.

[33] Grigsby-Toussaint D., Zenk S., Odoms-Young A., Ruggiero L., Moise I. (2010). Availability of commonly consumed and culturally specific fruits and vegetables in African-American and Latino neighborhoods. J. Am. Diet. Assoc..

