# Supplementary material for: Development of a Nutrition Environment Assessment Tool for Latino Ethnic Stores
Source: Int J Environ Res Public Health. 2022 Feb 7;19(3):1860. doi: 10.3390/ijerph19031860 (PMC8834718; doi:10.3390/ijerph19031860)

NEMS-S Latino Market Adaptation – Coversheet

Rater ID #: ______________________ Data Collection Date: _____/_____/_____

Field Check: ____________________ Data Entry: _____ Date: _____/_____/_____

Store Type: ◯ Grocery Store ◯ Ethnic Food Store ◯ Carnicería

◯ Convenience Store ◯ Other (Specify): __________________________

Type of Assessment: ◯ First ◯ Repeat/Second

Store Name: ____

Street Address: ____

City: _____________________________ State: _____ Zip Code: _____

Is the store currently in operation? ◯ YES ◯ NO

If the store is NOT in operation, is the building or store area…? (select all that apply)

◯ Closed ◯ Vacant ◯ Burned or Damaged

◯ Open for a different purpose (e.g. non-food store)?
If yes, specify: _________________________________

NOTE: **If store is closed, complete rest of coversheet. DO NOT MARK ON REMAINDER OF FORM. External evaluations of the store will be done after you complete the inside. Mark down the time before you proceed to enter the store:**

Store Assessment Start Time: ___ ___ **:** ___ ___ (use 24-hr clock)

Additional Comments (data collection team members):

____

____

NOTE: To complete the survey: Follow directions for reference item. If items not priced, write “NM” for “not marked.”

Store Name: ______________________ Rater ID #: _____

NEMS-S Latino Market Adaptation – Measure #1: Milk

Store sells white fluid milk? ◯ YES ◯ NO

NOTE: All items should be the same brand name if possible. If not possible, choose the Least Expensive Brand (LEB) or the brand with the most shelf space.

	Available?	Price	Carton Count / Comments
**1. Whole Milk (or Vitamin D Milk)** Brand: ________________________			
Gallon	◯ Yes ◯ No	$_______	____________________________
Half Gallon	◯ Yes ◯ No	$_______	____________________________
Quart	◯ Yes ◯ No	$_______	____________________________
Alternate: UHT*	◯ Yes ◯ No ◯ N/A	$_______	____________________________
**2. 2% Milk** Brand: ________________________			
Gallon	◯ Yes ◯ No	$_______	____________________________
Half Gallon	◯ Yes ◯ No	$_______	____________________________
Quart	◯ Yes ◯ No	$_______	____________________________
Alternate: UHT*	◯ Yes ◯ No ◯ N/A	$_______	____________________________
**3. Skim or 1% Milk** Brand: ________________________			
Gallon	◯ Yes ◯ No	$_______	____________________________
Half Gallon	◯ Yes ◯ No	$_______	____________________________
Quart	◯ Yes ◯ No	$_______	____________________________
Alternate: UHT*	◯ Yes ◯ No ◯ N/A	$_______	____________________________

*Ultra-High-Temperature (UHT) pasteurized milk available on the shelf or cooler sections.

NEMS-S Latino Market Adaptation –Measure #2: Eggs

Store sells any type of uncooked chicken eggs? ◯ YES ◯ NO

NOTE: The reference item for eggs is 1 dozen of LEB. If no price, choose eggs with highest carton count.

Available?	Price/Carton	Count/Carton	# Cartons Avail.	Comments
◯ Yes ◯ No	$ __________	____________	______________	____________________
Brand:	______________________________			

Number of brands of eggs (all types): ◯ 0 ◯ 1 ◯ 2 ◯ 3 ◯ 4 ◯ 5 ◯ 6+

Additional Comments: ____

Store Name: ______________________ Rater ID #: _____

Fruit and Vegetable Quality Measures
**Acceptable** = peak condition, top quality, good color, fresh, firm, clean<br>**Unacceptable** = bruised, old-looking, mushy, dry, overripe, dark sunken spots in irregular patches, cracked or broken surfaces, signs of shriveling, mold, excessive softening<br>**NOTE:** Record if "organic" options are available. If fruit is rated under 75% acceptable, comment why. Record additional varieties besides the referent fruit/vegetable.

NEMS-S Latino Market Adaptation – Measure #3: Fresh Fruit – Page 1 of 2

Store sells fresh fruit? ◯ YES ◯ NO

Produce Item	Available	Lowest Price	# Units	Per Unit	% Acceptable	Comments, record # of varieties w/in type
Bananas/Platanos<br>(regular yellow)	◯ Y<br>◯ N	$		◯ Piece<br>◯ Lb<br>◯ Size oz<br>__________	☐ ≤ 25% ☐ ≤ 50%<br>☐ ≤ 75 % ☐ >75%	
Apples/Manzanas<br>◯ Red Delicious<br>◯ __________________	◯ Y<br>◯ N	$		◯ Piece<br>◯ Lb<br>◯ Size oz<br>__________	☐ ≤ 25% ☐ ≤ 50%<br>☐ ≤ 75 % ☐ >75%	
Citrus<br>◯ Oranges/Naranjas<br>◯ Mandarins/Mandarina<br>◯ Grapefruit/Pomelo	◯ Y<br>◯ N	$		◯ Piece<br>◯ Lb<br>◯ Size oz<br>__________	☐ ≤ 25% ☐ ≤ 50%<br>☐ ≤ 75 % ☐ >75%	
Grapes/Uvas<br>◯ Red Seedless<br>◯ __________________	◯ Y<br>◯ N	$		◯ Piece<br>◯ Lb<br>◯ Size oz<br>__________	☐ ≤ 25% ☐ ≤ 50%<br>☐ < 75 % ☐ >75%	
Watermelon/Sandia<br>◯ Seedless<br>◯ __________________	◯ Y<br>◯ N	$		◯ Piece<br>◯ Lb<br>◯ Size oz<br>__________	☐ ≤ 25% ☐ ≤ 50%<br>☐ ≤ 75 % ☐ >75%	
Pears/Peras<br>◯ Anjou<br>◯ __________________	◯ Y<br>◯ N	$		◯ Piece<br>◯Lb<br>◯ Size oz<br>__________	☐ ≤ 25% ☐ ≤ 50%<br>☐ ≤ 75 % ☐ >75%	
Peaches/Duraznos	◯ Y<br>◯ N	$		◯ Piece<br>◯ Lb<br>◯ Size oz<br>__________	☐ ≤ 25% ☐ ≤ 50%<br>☐ ≤ 75 % ☐ >75%	
Melon<br>◯ Cantaloupe/Cantalupo<br>◯ Honeydew	◯ Y<br>◯ N	$		◯ Piece<br>◯ Lb<br>◯ Size oz<br>__________	☐ ≤ 25% ☐ ≤ 50%<br>☐ ≤ 75 % ☐ >75%	
Plantains/Platano	◯ Y<br>◯ N	$		◯ Piece<br>◯ Lb<br>◯ Size oz<br>_________	☐ ≤ 25% ☐ ≤ 50%<br>☐ ≤ 75 % ☐ >75%	

Store Name: ______________________ Rater ID #: _____

NEMS-S Latino Market Adaptation – Measure #3: Fresh Fruit – Page 2 of 2

Produce Item	Available	Lowest Price	# Units	Per Unit	% Acceptable	Comments, record # of varieties w/in type
Papaya<br>◯ Mexican<br>◯ _______________	◯ Y<br>◯ N	$		◯ Piece<br>◯ Lb<br>◯ Size oz<br>__________	☐ ≤ 25% ☐ ≤ 50%<br>☐ < 75 % ☐ >75%	
Mango (small yellow)<br>◯ Mexican (Ataulfo)<br>◯ _______________	◯ Y<br>◯ N	$		◯ Piece<br>◯ Lb<br>◯ Size oz<br>__________	☐ ≤ 25% ☐ ≤ 50%<br>☐ ≤ 75 % ☐ >75%	
Pineapple/Piña	◯ Y<br>◯ N	$		◯ Piece<br>◯ Lb<br>◯ Size oz<br>__________	☐ ≤ 25% ☐ ≤ 50%<br>☐ ≤ 75 % ☐ >75%	
Guava	◯ Y<br>◯ N	$		◯ Piece<br>◯ Lb<br>◯ Size oz<br>__________	☐ ≤ 25% ☐ ≤ 50%<br>☐ < 75 % ☐ >75%	
◯ Cactus Fruit/Tuna<br>◯ Xoconostle	◯ Y<br>◯ N	$		◯ Piece<br>◯ Lb<br>◯ Size oz<br>__________	☐ ≤ 25% ☐ ≤ 50%<br>☐ < 75 % ☐ >75%	

Total number of fresh fruits available: __________

Store Name: ______________________ Rater ID #: _____

NEMS-S Latino Market Adaptation – Measure #4: Fresh Vegetables – Page 1 of 2

Store sells fresh vegetables? ◯ YES ◯ NO

Produce Item	Available	Lowest Price	# Units	Per Unit	Percent Acceptable	Comments, # Varieties
Tomatoes/Tomato<br>◯ Roma<br>◯ Loose<br>◯ __________________	◯ Y<br>◯ N	$		◯ Piece<br>◯ Lb<br>◯ Size oz<br>__________<br>◯ Package	☐ ≤ 25% ☐ ≤ 50%<br>☐ ≤ 75 % ☐ >75%	
Lettuce/Lechuga<br>◯ Green Leaf<br>◯ Iceberg (only if no others)<br>◯ __________________	◯ Y<br>◯ N	$		◯ Piece<br>◯ Lb<br>◯ Size oz<br>__________	☐ ≤ 25% ☐ ≤ 50%<br>☐ ≤ 75 % ☐ >75%	
Carrots/Zanahoria<br>◯ 1 lb Bag<br>◯ ____________________	◯ Y<br>◯ N	$		◯ Piece<br>◯ Lb<br>◯ Size oz<br>__________<br>◯ Package	☐ ≤ 25% ☐ ≤ 50%<br>☐ ≤ 75 % ☐ >75%	
Cabbage/Repollo	◯ Y<br>◯ N	$		◯ Piece<br>◯ Lb<br>◯ Size oz<br>__________	☐ ≤ 25% ☐ ≤ 50%<br>☐ < 75 % ☐ >75%	
Cucumber/Pepino	◯ Y<br>◯ N	$		◯ Piece<br>◯ Lb<br>◯ Size oz<br>__________	☐ ≤ 25% ☐ ≤ 50%<br>☐ < 75 % ☐ >75%	
Corn/Elote	◯ Y<br>◯ N	$		◯ Piece<br>◯ Lb<br>◯ Size oz<br>__________	☐ ≤ 25% ☐ ≤ 50%<br>☐ < 75 % ☐ >75%	
Sweet Peppers<br>◯ Green Bell Pepper<br>◯ ____________________	◯ Y<br>◯ N	$		◯ Piece<br>◯ Lb<br>◯ Size oz<br>__________<br>◯ Package	☐ ≤ 25% ☐ ≤ 50%<br>☐ < 75 % ☐ >75%	
Celery/Apio	◯ Y<br>◯ N	$		◯ Piece<br>◯ Lb<br>◯ Size oz<br>__________	☐ ≤ 25% ☐ ≤ 50%<br>☐ < 75 % ☐ >75%	
Tomatillos<br>◯ Small<br>◯ Large	◯ Y<br>◯ N	$		◯ Piece<br>◯ Lb<br>◯ Size oz<br>__________	☐ ≤ 25% ☐ ≤ 50%<br>☐ < 75 % ☐ >75%	

Store Name: ______________________ Rater ID #: _____

NEMS-S Latino Market Adaptation – Measure #4: Fresh Vegetables – Page 2 of 2

Produce Item	Available	Lowest Price	# Units	Per Unit	Percent Acceptable	Comments, # Varieties
Chiles<br>◯ Anaheim<br>◯ Poblano<br>◯ Pasilla	◯ Y<br>◯ N	$		◯ Piece<br>◯ Lb<br>◯ Size oz<br>__________	☐ ≤ 25% ☐ ≤ 50%<br>☐ ≤ 75 % ☐ >75%	
Squash/Calabaza<br>◯ Calabacita/Zucchini<br>◯ Chayote<br>◯ __________________	◯ Y<br>◯ N	$		◯ Piece<br>◯ Lb<br>◯ Size oz<br>__________	☐ ≤ 25% ☐ ≤ 50%<br>☐ ≤ 75 % ☐ >75%	
Avocado/Aguacate<br>◯ Hass (bumpy skin)<br>◯ Fuerte (smooth skin)	◯ Y<br>◯ N	$		◯ Piece<br>◯ Lb<br>◯ Size oz<br>__________	☐ ≤ 25% ☐ ≤ 50%<br>☐ ≤ 75 % ☐ >75%	
Radishes/Rabanos	◯ Y<br>◯ N	$		◯ Piece<br>◯ Lb<br>◯ Size oz<br>__________	☐ ≤ 25% ☐ ≤ 50%<br>☐ < 75 % ☐ >75%	
Jicama	◯ Y<br>◯ N	$		◯ Piece<br>◯ Lb<br>◯ Size oz<br>__________	☐ ≤ 25% ☐ ≤ 50%<br>☐ < 75 % ☐ >75%	
Cactus Pad/Nopal	◯ Y<br>◯ N	$		◯ Piece<br>◯ Lb<br>◯ Size oz<br>__________	☐ ≤ 25% ☐ ≤ 50%<br>☐ < 75 % ☐ >75%	
Yucca	◯ Y<br>◯ N	$		◯ Piece<br>◯ Lb<br>◯ Size oz<br>__________	☐ ≤ 25% ☐ ≤ 50%<br>☐ < 75 % ☐ >75%	
Onion/Cebolla<br>◯ White Onion<br>◯ __________________	◯ Y<br>◯ N	$		◯ Piece<br>◯ Lb<br>◯ Size oz<br>__________	☐ ≤ 25% ☐ ≤ 50%<br>☐ < 75 % ☐ >75%	
Potatoes/Papas (brown skin, white flesh)	◯ Y<br>◯ N	$		◯ Piece<br>◯ Lb<br>◯ Size oz<br>__________	☐ ≤ 25% ☐ ≤ 50%<br>☐ < 75 % ☐ >75%	
Sweet Potato	◯ Y<br>◯ N	$		◯ Piece<br>◯ Lb<br>◯ Size oz<br>__________	☐ ≤ 25% ☐ ≤ 50%<br>☐ < 75 % ☐ >75%	

Total number of fresh vegetables available: __________

Store Name: ______________________ Rater ID #: _____

NEMS-S Latino Market Adaptation – Measure #5A: Beefsteak/Ground Beef

Store sells fresh or frozen beef/beefsteak? ◯ YES ◯ NO

Healthier Option:
1. Lean Beef (≤ 10% fat)? ◯ YES ◯ NO
(e.g., Milanesa de Pulpa Bola, Pulpa Negra, Arrachera)

Regular Option:
2. Regular Beef (> 10% fat)? ◯ YES ◯ NO
(e.g. Pulpa Larga, Costillas Cargadas)

NEMS-S Latino Market Adaptation – Measure #5B: Chicken/Fish

Store sells fresh or frozen chicken? ◯ YES ◯ NO

Item	Available	Price/lb	Comments
**Healthier Option:**			
1. Fresh/Frozen Chicken Breast or White Meat/Pechuga de Pollo	◯ Yes ◯ No	$_______	____________________
Skinless?	◯ Yes ◯ No ◯ N/A		
**Regular Option:**			
2. Fresh/Frozen Legs & Thighs (dark meat)/Piernas y Muslos	◯ Yes ◯ No	$_______	____________________
Skinless?	◯ Yes ◯ No ◯ N/A		

NEMS-S Latino Market Adaptation – Measure #5C: Fish

1. Store sells fresh or frozen fish? ◯ YES ◯ NO

2. Total number of varieties of fresh & frozen fish available: __________

NOTE: Exclude seafood: shrimp, octopus, crab, calamari, etc. Do not count the same fish type twice if available in different forms.

Store Name: ________________________ Rater ID #: _____

NEMS-S Latino Market Adaptation – Measure #6: Beverages

Beverage Item	Available	Size (oz)	Price	Packaging	Comments
[1] Healthier Option: Water, Brand:_____________________	◯ Y<br>◯ N		$	◯ Plastic<br>◯ Glass	
[1] Alternate Option: Topo Chico Mineral Water	◯ Y<br>◯ N<br>◯ N/A		$	◯ Plastic<br>◯ Glass	
[2] Healthier Option: Minute Maid 100% Juice	◯ Y<br>◯ N		$	◯ Plastic<br>◯ Glass	
[2] Alternate Option: Other Brand 100% Juice, Brand: ___________________	◯ Y<br>◯ N<br>◯ N/A		$	◯ Plastic<br>◯ Glass	
[3] Regular Option: Coke (12 oz can)	◯ Y<br>◯ N	12 oz	$	Can	
[3] Coke (20 oz plastic)	◯ Y<br>◯ N	20 oz	$	Plastic	
[3] Coke (12 oz/355 mL glass)	◯ Y<br>◯ N	12 oz	$	Glass	
[3] Coke (16.9 oz/half liter glass)	◯ Y<br>◯ N	16.9 oz	$	Glass	
[3] Alternate Option: Other Sugared Soda (12 oz), Brand: ___________________	◯ Y<br>◯ N<br>◯ N/A	12 oz	$	◯ Can<br>◯ Glass	
[3] Other Sugared Soda (20 oz), Brand: ___________________	◯ Y<br>◯ N<br>◯ N/A	20 oz	$	◯ Plastic<br>◯ Glass	
Regular Option: Jumex Pineapple Nectar (40% Juice, 11.3 oz/355 mL)	◯ Y<br>◯ N	11.3 oz	$		
Alternate Option: Jumex Pineapple Nectar (Other Size)	◯ Y<br>◯ N<br>◯ N/A		$		

[1] **Water:** Look in the cooler first for beverages. Look for 16.9 oz water first. If not available, find next smallest size. If no regular bottled water available, look for mineral water.
[2] **Juice:** Look for juice between 8-20 oz. If not available, look for next smallest size. Do not count V-8. Orange=preferred, apple=alternate.
[3] **Soda:** Look for 12 oz and 20 oz Coke in can and plastic bottle, as well as Coke in glass bottles (typically 355 mL and half liter).

Store Name: ________________________ Rater ID #: _____

NEMS-S Latino Market Adaptation – Measure #7: Tortillas

Healthiest Option: Corn (Yellow & White)

1. Store sells corn tortillas? ◯ YES ◯ NO

2. Number of brands of corn tortillas ◯ 0 ◯ 1 ◯ 2 ◯ 3 ◯ 4 ◯ 5 ◯ 6+

Comments: ____

Healthier Option: Whole Wheat Flour Tortillas (100% Whole Wheat or Whole Grain)

1. Stores sells whole wheat flour tortillas? ◯ YES ◯ NO

2. Number of brands of whole wheat tortillas ◯ 0 ◯ 1 ◯ 2 ◯ 3 ◯ 4 ◯ 5 ◯ 6+

Comments: ____

Regular Option: Flour Tortillas (Made with Refined Flour)

1. Store sells flour tortillas? ◯ YES ◯ NO

2. Number of brands of flour tortillas ◯ 0 ◯ 1 ◯ 2 ◯ 3 ◯ 4 ◯ 5 ◯ 6+

Comments: ____

NEMS-S Latino Market Adaptation – Measure #8: Canned Beans – Page 1 of 2

NOTE: Choose the smallest size available in the referent brand of beans.

Healthiest Option: Low Sodium Whole Canned Beans

1. Store sells low sodium canned beans? ◯ YES ◯ NO

If yes, are they… (select all that apply): ◯ Pinto ◯ Black ◯ Red
◯ Other: _______________________

2. Number of brands of low sodium canned beans: ________

Healthier Option: Whole Canned Beans

	Available?	Price	Size (oz)
1. La Preferida Whole Pinto Beans	◯ Y ◯ N	$__________	__________
	Comments: ____________________		
**Alternate Items:**			
2. La Costeña Whole Pinto Beans	◯ Y ◯ N ◯ N/A	$__________	__________
	Comments: ____________________		
3. Whole Black Beans	◯ Y ◯ N ◯ N/A	$__________	__________
Brand: ____________________	Comments: ____________________		

Baier-Winham Latino Market NEMS-S; dwinham@iastate.edu

Store Name: ______________________ Rater ID #: _____

NEMS-S Latino Market Adaptation – Measure #8: Canned Beans -- Page 2 of 2

4.What other brands of whole canned beans are available? (select all that apply)

◯La Preferida	◯La Costeña	◯Goya	◯El Gallito	◯Ducal	◯El Mexicano	◯La Sierra
◯Bush Bros.	◯Teasdale	◯La Criola	◯La Sierra	◯San Marcos	◯Malher	◯Adelita
◯Tio Q	◯Nautra	◯La Morena	◯Other: ______________________________			

5. Number of brands of whole canned beans (not including refried): __________

---

6. Regular Option: Refried Beans

	Available?	Price	Size (oz)
1. La Preferida Refried Pinto Beans	◯ Y ◯ N	$__________	__________
	Comments: ______________________________		
**Alternate Items:**			
2. La Costeña Refried Pinto Beans	◯ Y ◯ N	$__________	__________
	◯ N/A		
	Comments: ______________________________		
3. Refried Black Beans	◯ Y ◯ N	$__________	__________
	◯ N/A		
Brand: ______________________	Comments: ______________________________		

7. Number of brands of canned refried beans (include pinto, black, and red refried): __________

8. Store sells 98-100% fat-free refried beans? ◯ YES ◯ NO

If yes, what brands? ______________________________

Store Name: _______________________ Rater ID #: _____

NEMS-S Latino Market Adaptation – Measure #9: Dry Beans

NOTE: Choose the LEB in the 1 lb (16oz) bag. If packaged by store into smaller bags, note "store self-packaged" under comments, and write in the price per lb. If no size is indicated, write "NM" by lb for "not marked."

	Yes	No	N/A	Price	Brand/Comments
1. Pinto Beans, 1 lb	◯	◯		**$**_________	____________________________
**Alternate Item:**					
Pinto Other Size, _____ lb	◯	◯	◯	$_________	____________________________
2. Black Beans, 1 lb	◯	◯		**$**_________	____________________________
**Alternate Item:**					
Black Other Size, _____ lb	◯	◯	◯	$_________	____________________________
3. Mayocoba Beans, 1 lb.	◯	◯		**$**_________	____________________________
**Alternate Item:**					
Mayocoba Other Size,_____ lb	◯	◯	◯	$_________	____________________________
4. Red Beans, 1 lb	◯	◯		**$**_________	____________________________
**Alternate Item:**					
Red Other Size, _____ lb	◯	◯	◯	$_________	____________________________

Store Name: _______________________ Rater ID #: _____

NEMS-S Latino Market Adaptation – Measure #10: Rice

NOTE: Referent is 1 lb (16 oz) bag/box of LEB. If not available in 1 lb, look for next smallest size available.

	Yes	No	N/A	Price	Brand/Comments
**Healthier Option:**					
1. Brown Rice	◯	◯		$_________	____________________________
Brand: __________________					
**Alternate Option:**					
2.White Parboiled Rice	◯	◯	◯	$_________	____________________________
Brand: __________________					
**Regular Option:**					
3. White Large Grain Rice	◯	◯		$_________	____________________________
Brand: __________________					

Store Name: ______________________ Rater ID #: __________

NEMS-S Latino Market Adaptation – Measure #11: Canned Fruits and Vegetables

NOTE: Select brand based on shelf space for cans/jars sized between 14-16 oz. If not available, choose smallest size available of the LEB.

**Canned Fruits**	**Avail?**	**Price**	**Can/Jar Size (oz)**	**Packaging**	**Comments**
Pineapple	◯ Y<br>◯ N	$		☐ Water<br>☐ Light Syrup<br>☐ 100% Juice<br>☐ Heavy Syrup	
Guava	◯ Y<br>◯ N	$		☐ Water<br>☐ Light Syrup<br>☐ 100% Juice<br>☐ Heavy Syrup	
Cherries (yellow Nance or other – *look for glass*)	◯ Y<br>◯ N	$		☐ Water<br>☐ Light Syrup<br>☐ 100% Juice<br>☐ Heavy Syrup	
Mango	◯ Y<br>◯ N	$		☐ Water<br>☐ Light Syrup<br>☐ 100% Juice<br>☐ Heavy Syrup	
Fruit Cocktail	◯ Y<br>◯ N	$		☐ Water<br>☐ Light Syrup<br>☐ 100% Juice<br>☐ Heavy Syrup	
Peaches	◯ Y<br>◯ N	$		☐ Water<br>☐ Light Syrup<br>☐ 100% Juice<br>☐ Heavy Syrup	
**Canned Vegetables**	**Avail?**	**Price**	**Can/Jar Size (oz)**	**Packaging**	**Comments**
Corn (kernel)	◯ Y<br>◯ N	$		☐ Low Sodium<br>☐ Regular Pack	
Green Peas (NOT pigeon peas)	◯ Y<br>◯ N	$		☐ Low Sodium<br>☐ Regular Pack	
Nopalitos	◯ Y<br>◯ N	$		☐ Low Sodium<br>☐ Regular Pack	
Mixed Vegetables	◯ Y<br>◯ N	$		☐ Low Sodium<br>☐ Regular Pack	
Green Beans	◯ Y<br>◯ N	$		☐ Low Sodium<br>☐ Regular Pack	
Tomatillos	◯ Y<br>◯ N	$		☐ Low Sodium<br>☐ Regular Pack	

Total number of canned fruits available: __________

Total number of canned vegetables available: __________

Store Name: ______________________ Rater ID #: __________

NEMS-S Latino Market Adaptation – Measure #12: Canned Fish

NOTE: Look for size indicated and choose the LEB. If referent size not available find the next smallest size.

Store sells canned fish? ◯ YES ◯ NO

	Avail?	Price	Brand/Comments
Sardines in Tomato Sauce (15 oz)	◯ Y ◯ N	$	
Sardines in Water (15 oz)	◯ Y ◯ N	$	
Sardines in Oil (15 oz)	◯ Y ◯ N	$	
Tuna in Water (5 oz)	◯ Y ◯ N	$	
Tuna in Oil (5 oz)	◯ Y ◯ N	$	

NEMS-S Latino Market Adaptation – Measure #13: Cooking Fats/Oils

NOTE: Record the price of 16 oz (473 mL) or the next smallest size. For solid fats, use 1 lb (16 oz) as the reference.

1. Store sells cooking oils? ◯ YES ◯ NO

2. Types available? ◯ Corn Oil ◯ Canola Oil ◯ Extra Virgin Olive Oil ◯ Olive Oil ◯ Vegetable Oil

3. Number of types of cooking oils available: ◯ 0 ◯ 1 ◯ 2 ◯ 3 ◯ 4 ◯ 5 ◯ 6+

----

1. Store sells solid fats? ◯ YES ◯ NO

2. Types available? ◯ Lard (Manteca) ◯ Vegetable Shortening ◯ Butter/Margarine

3. Number of types of solid fats available: ◯ 0 ◯ 1 ◯ 2 ◯ 3 ◯ 4+

Additional Comments:

____

____

Before leaving the store, record the:

Number of cash registers: __________

Number of aisles: _________

Does this store have a freezer or frozen food section?	◯ YES	◯ NO
Does this store have a meat counter?	◯ YES	◯ NO
Is this store also a restaurant?	◯ YES	◯ NO
Does this store sell alcohol?	◯ YES	◯ NO
Does this store sell cigarettes?	◯ YES	◯ NO
Does the manager say that the store accepts WIC vouchers?	◯ YES	◯ NO
Does the manager say that the store accepts SNAP/EBT?	◯ YES	◯ NO

Store Assessment End Time Inside: ___ ___ : ___ ___ (use 24-hr clock)

NOTE: Remember to note the time you finished and recheck your work before leaving the store! Thank any staff and/or manager that helped you.

Complete observations of the store's external environment – are any of these services available outside of the store?

Is there an outside sign or decal that says the store accepts WIC vouchers?	◯ YES	◯ NO
Is there an outside sign or decal that says the store accepts SNAP/EBT?	◯ YES	◯ NO
Is there an outside sign or decal that says the store sells tobacco products?	◯ YES	◯ NO
Is there an outside sign or decal that says the store sells alcoholic beverages?	◯ YES	◯ NO

Were photographs taken of exterior of store?	◯ YES	◯ NO

Number of photographs taken: __________

Double check your work for completeness, accuracy, and legibility before you leave the parking lot.

Latino NEMS–S Data Collection Protocol

December 29, 2021
J.L. Baier & D.M. Winham
dwinham@iastate.edu
Iowa State University

When do I do the survey?

- Try to complete the survey between the hours of 9:00 am and 4:00 pm (some stores may not open until 10:00 or 11:00 am). This helps ensure that items are in stock and have not sold out.
- It is estimated to take up to 60 minutes to complete the survey.
- Make sure you move out of the way of customers or store employees during your assessment. It is essential to be as unobtrusive as possible while thoroughly completing the form.

Outside the Store: What do I need to do before I go in?

- Before you go into the store, fill out the bulk of the cover page while in your car or the parking lot.
- Ensure the store name and address are correct.
- Briefly review the form as a reminder of what you will be looking for during your visit. Note if you find it difficult to assess certain items.
- Photos of items taken with your phone are welcome to document your observations, but be cautious as storeowners may be uncomfortable with you taking pictures in their store. Best to ask permission. If taking pictures of the outside of the store, take them on the way out after the survey has been completed.

Inside the Store: What do I do once I am inside?

- **Introduce yourself and obtain consent**
    - Introduce yourself to an available clerk or ask to see the store manager. Explain who you are and what you are doing using the script provided. You should give enough information for a high-level understanding of the purpose of your visit.
    - Provide the clerk or manager a copy of your agency's letter of explanation. Stores should also have received an introductory letter in the mail.
    - Permission to conduct the survey must be obtained before data collection begins.
    - If you are visiting a store that has previously been assessed, explain to the staff that this store participated in the past and that you are doing a follow-up survey.
- **Conducting the survey**
    - The store manager is likely to consent and be compliant. However, if asked to leave at any point during the survey, thank them for their time and promptly leave.
    - Be courteous and professional at all times.
    - Always make an effort to avoid being in the way of customers or store personnel. We do not want to interfere with the normal flow of business in any way.
    - Leave store shelves as you found them, returning items to their place after collecting the necessary information.

The Latino NEMS-S Survey Measures Procedure

- The measures in the NEMS survey often have multiple components. Work carefully to ensure you do not skip a section or step. The basic process for each measure is as follows:

1. Look for a Regular Option and a Healthier Option of the item listed.
    - If the ideal Regular Option or Healthier Option is unavailable, record data for the specified Alternate Option.
    - Use the same brand for both the Regular and Healthier Options if possible.

2. If the reference brand is unavailable:
    - Use the guidelines provided on the form. This will determine whether to use the item that is the correct size, takes up the most shelf space, or is the Least Expensive Brand (LEB).
    - Utilize the comments section to further describe the alternate product (brand, size, etc).

3. If the item appears to be typically sold, but is out of stock, write "Sold Out."

4. If the reference item is available, there is no need to look for the Alternate Option. Mark the circle for N/A (not applicable).

5. Pricing:
    - Always record the regular price (not the sale price).
      If there is no price listed, complete the rest of the survey, then ask the clerk or manager if they can tell you the missing prices of selected items (note it is not practical to ask for prices on more than 5-7 items). No prices are particularly common for fresh fruits and vegetables.

6. Note any discrepancies or items that are difficult to assess (e.g. an item on the shelf does not match any of the size options on the form). When in doubt, make notes so there is a clear record of your findings.

Tips and Reminders for Specific Measures

The next section includes instructions and tips that may help with the unique circumstances of each store, and for the additional measures we are testing (beefsteak, chicken, fish, tortillas, bottled water, nectars, canned beans, dried beans, rice, oils and fats, canned fruits and vegetables).

Measure #1 – Milk:

Latino stores often have a small amount of milk. Count the total number of containers for each type of milk available.

1. Look for white milk (not chocolate or other flavored milk).

2. If you do not see fluid milk in the coolers, look for the alternate cartons of ultra-high-temperature (UHT) processed milk. These are shelf-stable and do not require refrigeration (though stores might still stock it in coolers).

3. Ask for the price at the end of your visit if it is not marked.

Measure #2 – Eggs:

1. Eggs are usually found near the milk in coolers. Record the information for the "store" brand or the LEB carton of one dozen eggs that are grade A or AA, size large, and white. Write down the brand name, how many eggs there are per carton, and the number of cartons available in the cooler.

2. Mark the total number of different brands of eggs available, including brown eggs.

Measures #3-4 – Fresh Fruits & Vegetables:

1. Keep in mind that fresh fruit and vegetable availability will vary based on store location and season. The original NEMS-S instructions has more information on classifying quality of the fresh fruits and vegetables if needed.

2. % Acceptable: This should be based on the quality of the majority of each fruit item. If less than 75% acceptable, record why in the comments section (e.g. bruised, moldy spots).

3. Price, # Units, and Per Unit: Mark the lowest price, the number of units included for that price, and how a unit is defined. For example, if the sign says "$1.99 for 3 lbs," write *1.99* as the "price," *3* as "# units," and mark *lbs* under "per unit."

4. Location: Fresh fruits are typically stocked together in one section of the store, however there may be one or two fruits that are located close to the registers. The fresh fruits may be stocked or in boxes on the floor.

5. Choosing the right fruits and vegetables to measure:
   - Verify which items are the reference type. Use the glossary for reference if needed in identification of fruits and vegetables.
   - Record the total number of varieties of a particular fruit or vegetable. For example, Red Delicious apples are the reference apple, but a store might also stock Granny Smith, Macintosh, and Fuji apples. Under the comments section for apples, you would note 4 varieties and list the names.
   - Pre-Sliced Fruits and Vegetables: Mark "yes" for available and write "pre-cut" and any size or package information in the comments.

6. What to look for with specific vegetables:
   - Chiles: Look for chiles that are used in entrees, not just as condiments. The preferred chile is Anaheim and acceptable alternatives are poblanos and pasillas. Note that smaller jalapeños and serranos should not be recorded as vegetables for this purpose.
   - Carrots: Look for whole, regular size carrots in a 1 lb package. Some markets will have very large single or multiple packed carrots. Make a note describing these if found. Only select baby or pre-cut carrots as a last resort and note in comments.

- Tomatoes: Look for the least expensive Roma tomatoes first. If not available, look for loose tomatoes. Choose tomatoes on the vine or cherry tomatoes as a last resort and note in comments.
- Broccoli: Look for the bunch (including longer stem) before the smaller crowns.
- Cactus/Nopal: Look for cactus pad first, then the aloe.
- Tomatillos: These may be available in small and larger size. If both are available, choose the smaller size first.

7. Include the number of different varieties in comments:
   - For fruits and vegetables, count the number of different types or varieties for each item. Do not enter different varieties as additional fruits or vegetables in the spaces designated for other items.
   - For example, red grapefruit is the reference brand. If the store also has white grapefruit, record it as a second variety under comments. Do not count it as a different fruit.

Measure #5 – Beefsteak, Chicken, & Fish:

1. Beef and Chicken: Note the price per pound only rather than the total price on the package label if available. You may ask the butcher if prices are not listed.

2. Beefsteak: Cuts may vary by store. You are looking for the lean type of beef that would be used to make milanesa. Ground beef can also be included. Although many tiendas will grind it on order.

3. Do not include breaded or whole chicken. Record whether chicken is available, and also whether a skinless option is available. Look for fresh chicken first, then frozen.

4. Fish: These may be in fillet or whole fish form. Fish may be located at the meat counter and/or in different coolers around the store. Common types are tilapia and mojarra. Do not count one type of fish as two if it is available fresh and frozen or in different cuts. Record the total number of varieties of fish. Frozen fish might not have any identifying information.

Measure #6 – Beverages/Juice:

1. In this measure, you will look for soda, juice, water, and juice nectar. Make sure to ask the store manager for price if it is not marked. Note we are assessing three levels of beverage "healthiness" – 100% juice, about 40% juice, and no juice (e.g. soda).

2. For water and juice, the form will ask you to mark whether the beverage is packaged in plastic or glass containers.

3. Juice: Look for Minute Maid 100% orange juice first. If this brand is not available, choose the juice with the most shelf space that is between 8-20 oz. If this size is not available, choose the next smallest size of 100% juice available. Do not count V-8.

4. Soda: Look for 12 oz for aluminum can and glass, 20 oz in plastic, and 16.9 oz glass. The 16.9 oz (or half liter) glass is the size most commonly found that is closest to 20 oz. If the

not available, look for an alternate brand of sugared soda (non-diet) that has the most shelf space.

5. Water: Look for 16.9 oz bottled water. If not available, look for next smallest size of water. If no regular water available, look for mineral water.

6. Fruit Drinks/Nectars: The healthy option is pineapple nectar as it has the highest % of juice of the nectars. Look for the 11.3 oz can. If not available, use the next smallest size. Nectars are typically located in more than one area of the store. They may be stocked on shelves, in coolers, or at the front of the store.

Measure #7 – Tortillas:

1. Record whether corn, whole wheat, and flour tortillas are available in the store and fill in the number of brands available. Note that flour tortillas can come in small packages and be confused with corn tortillas.

2. Tortillas will likely be stored in coolers but can also be located on shelves and endcaps. These sections may also contain different brands than the tortillas in the refrigerated section.

Measure #8 – Canned Beans:

1. In this measure, you will look for Fat-Free Refried Beans, Regular Refried Beans, Whole Canned Beans, and Low Sodium Whole Canned Beans.

2. Look for the La Preferida brand of beans first. If not available, look for the La Costena brand. If the only type of beans available are flavored pinto, try to choose “Authentic” or original versions over other specialty flavors.

Measure #9 – Dry Beans:

1. The preferred size is a 1 lb (16 oz) bag (no specific brand). If not available, look for a 2 lb package in the most common brand. If not available, look for bulk containers of the specified bean, or the next smallest size.

2. Select the LEB of beans in the specified size.

3. If no common brand, record the brand names of the beans that are available in 1 lb packages.

4. There may be clear, store-packaged unmarked bags of dry beans available. These will not be included as the preferred options but are considered an alternate option if marked bags of dry beans are not available.

Measure #10 Rice:

1. For this measure you are looking for 1 lb (16 oz) bags/boxes of brown rice and white rice (parboiled, long grain).

2. Look for the LEB in 1 lb (16 oz). If not available, look for the next smallest available size.

Measure #11 – Canned Fruits/Vegetables:

1. For this measure you are looking for canned or jarred fruits and vegetables. Look for sizes between 14-16 oz (or the next smallest available size).

2. Check the appropriate box to indicate what liquid the fruit is packaged in. This is typically written on the front of the can. Also note if the vegetables are available with low/reduced sodium.

Measure #12 – Canned Fish:

1. Look for the referenced size of the fish and find the LEB in that size. If the fish is not available as indicated, note in the comments the size and brand of the available product.

Measure #13 – Cooking Oils/Solid Fats:

1. The reference is 16 oz (473 mL), or the next smallest size available of each of the oils. Mark the total types of oils available.
2. Look for 16 oz of solid fats (lard, shortening, butter/margarine). If not available, look for next smallest available size. Mark the total types of solid fats available.

Before Leaving the Store:

1. Write down the number of cash registers. Include any at the pharmacy, deli, customer service, and the "manned" cash register in the self-checkout area. Each cash register should be counted even if a clerk is not physically present at the time of your visit.
2. Make sure to complete the last page of the form.
3. <u>Remember to thank the store manager before leaving!</u>

Outside of the Store:

- Look for advertisements on the building itself.
- Look for signs or decals that say the store accepts WIC vouchers/SNAP/EBT.
- Look to see if there is a sign that says the stores sells alcoholic beverages.
- Take pictures of exterior of the store.
- Note the end time.

Before You Submit Your Surveys:

- Ensure your rater ID and/or name is on the form.
- Ensure the form for each store is complete.

Latino NEMS-S Scoring Guide
Baier et al., 2022; dwinham@iastate.edu

	Availability	Quality
1. **Milk (3)**	Milk Available = 2 pts<br>Low Fat or 1% = 1 pt<br>No Milk Sold = 0 pts	
2. **Eggs (2)**	Eggs Available = 1 pt<br>• >1 Brand = Additional 1 pt<br>No Eggs Sold = 0 pts	
3. **Fresh Fruits (6)**	Fresh Fruit Available = 1 pt<br>>9 Varieties = 2 pts<br>5-9 Varieties = 1 pt<br>No Fresh Fruits Sold = 0 pts	>75% Acceptable = 3 pts<br>50-75% Acceptable = 2 pts<br><50% Acceptable = 1 pt<br>≤25% Acceptable = 0 pts
4. **Fresh Vegetables (6)**	Fresh Vegetables Available = 1 pt<br>>9 Varieties = 2 pts<br>5-9 Varieties = 1 pt<br>No Fresh Vegetables Sold = 0 pts	>75% Acceptable = 3 pts<br>50-75% Acceptable = 2 pts<br><50% Acceptable = 1 pt<br>≤25% Acceptable = 0 pts
5. **Fresh Beefsteak (3)**	Lean Option = 2 pts<br>Regular Option = 1 pt<br>No Beefsteak/Ground Beef Sold = 0 pts	
6. **Fresh/Frozen Chicken (4)**	Lean Option = 2 pts<br>Regular Option = 1 pt<br>• Skinless Option = Additional 1 pt<br>No Chicken Sold = 0 pts	

7. **Fresh/Frozen Fish (2)**
Fresh/Frozen Fish Available = 2 pts
No Fish Sold = 0 pts

8. **Beverages (5)**
Bottled Water = 2 pts
100% Juice = 2 pts
Fruit Nectar = 1 pt
Regular Soda = 0 pts

9. **Tortillas (4)**
Tortillas Available = 1 pt
Corn Tortillas = 2 pts
Whole Wheat Tortillas = 1 pt

10. **Canned Beans (5)**
Whole Canned Beans = 2 pts
- Low Sodium = Additional 1 pt

Regular Refried Beans = 1 pt
≥4 Brands Whole Canned Beans = 1 pt

11. **Dry Beans (2)**
Dry Beans Available = 1 pt
≥3 Varieties = 1 pt

12. **Rice (3)**
Brown/Parboiled Rice Available = 2 pts
White Rice = 1 pt

13. **Canned Fruits (3)**
>4 Varieties = 2 pts
1-4 Varieties = 1 pt
- 3+ Fruits in Water/100% Juice = Additional 1 pt

0 Varieties Sold = 0 pts

14. **Canned Vegetables (3)**
>4 Varieties Available = 2 pts
1-4 Varieties = 1 pt
- 3+ Vegetables in Low Sodium/Water = Additional 1 pt

0 Varieties Sold = 0 pts

15. **Canned Fish (3)**
Canned Fish Available = 2 pts
- ≥2 Packaging Varieties (e.g. Oil, Tomato Sauce, Water) = Additional 1 pt

16. **Cooking Oils (3)**
Fluid Oil Available = 2 pts
- ≥3 Fluid Oils = Additional 1 pt

Food Item	Preferred Option	Alternative Option
**Apple**	Red Delicious: 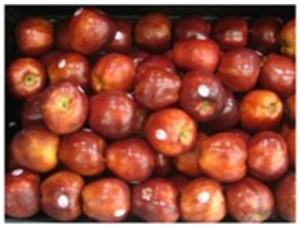 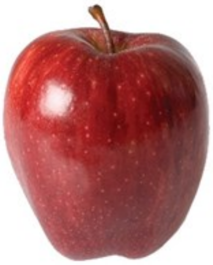	Least Expensive Apple
**Orange**	Navel Orange: 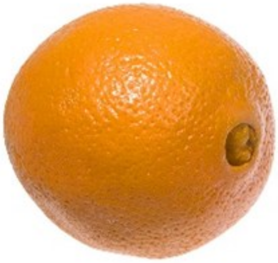	Valencia Orange: 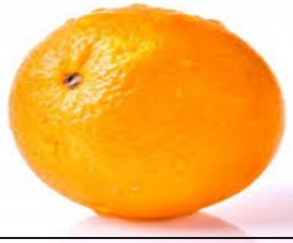
**Cantaloupe**	Whole Cantaloupe: 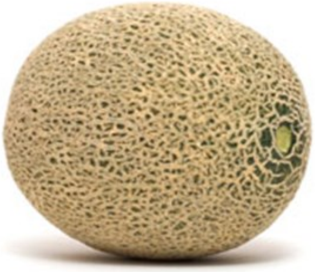	Honeydew Melon: 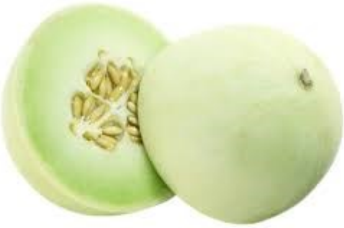
**“Cactus Fruit”/Tuna**	Tuna: 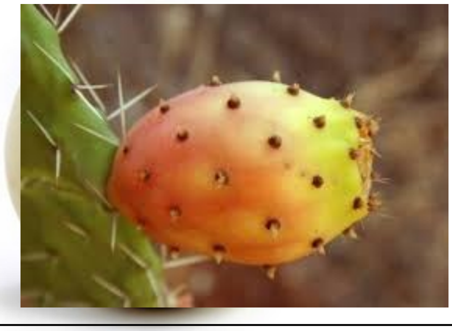	Xoconostle: 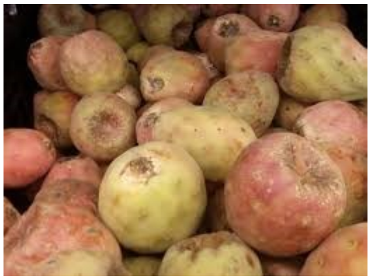
**Pear**	Anjou Pear: 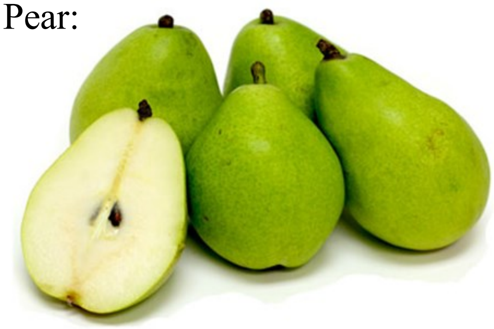	No Alternative
**Grapefruit**	Red Grapefruit: 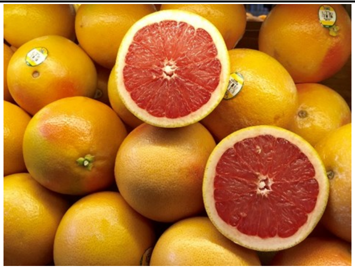	No Alternative

**Papaya**	Mexican Papaya: 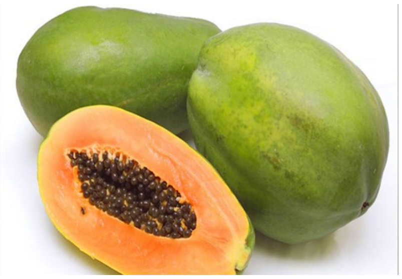 When Mexican papaya is ripe, it often has soft spots and blemishes on the skin. 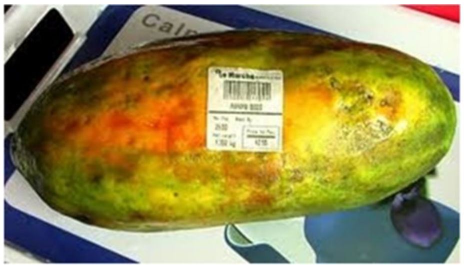	Hawaiian Papaya: 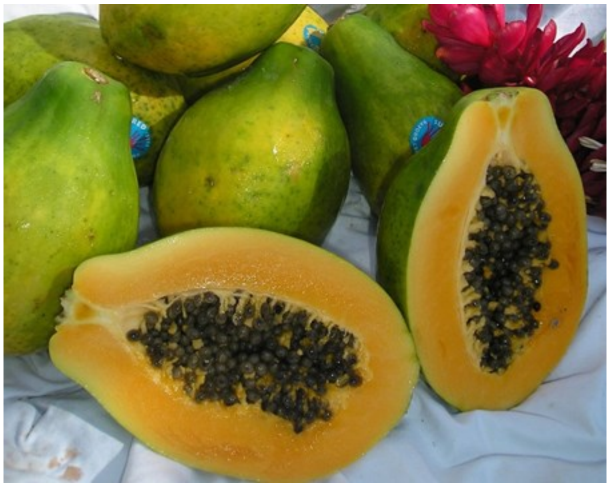
**Mango**	Ataulfo Mango: 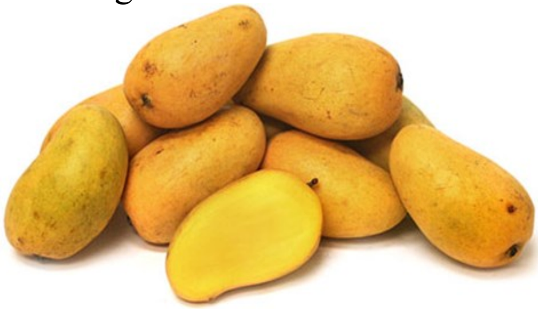	Tommy Perkins Mango: 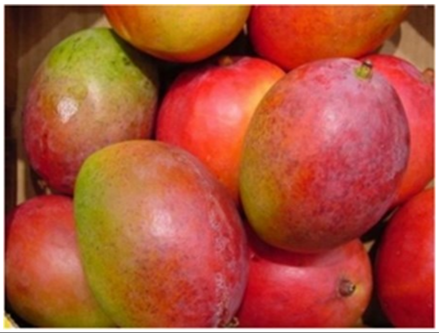
**Guava**	Common Guava: 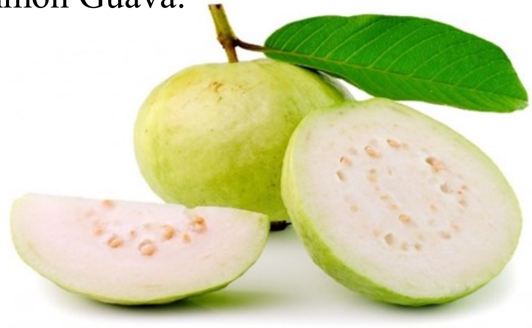	No Alternative
**Tomatoes**	Roma Tomatoes: 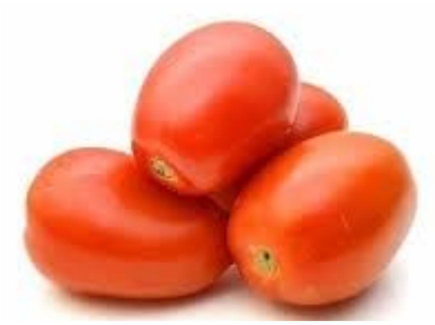	Loose Tomatoes: 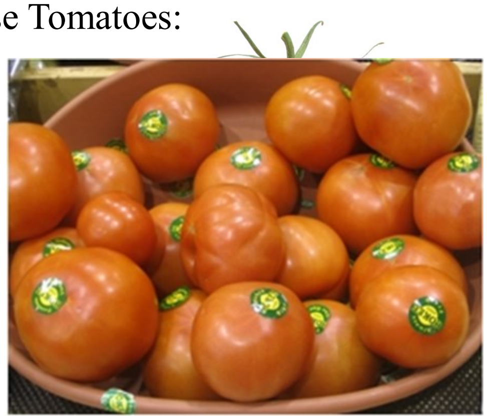

**Lettuce**	Green Leaf Lettuce: 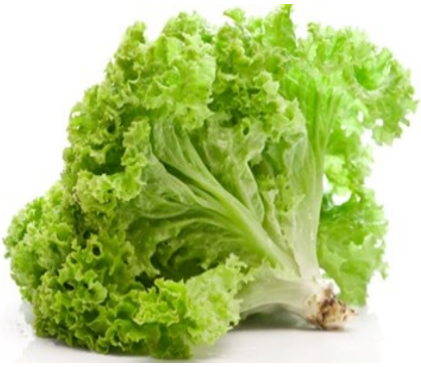	Iceberg Lettuce: 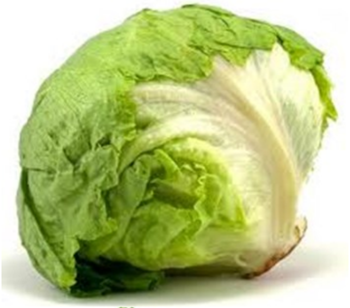 Romaine Lettuce: 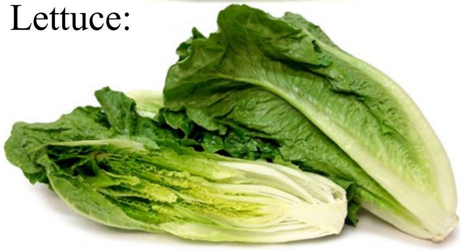
**Avocado**	Hass Avocado: 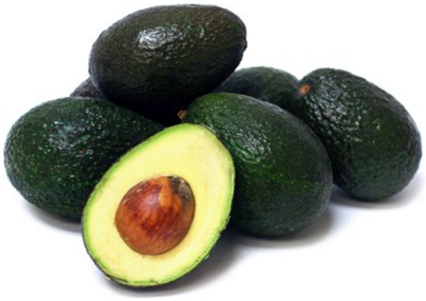	Fuerte Avocado (Smooth Skin): 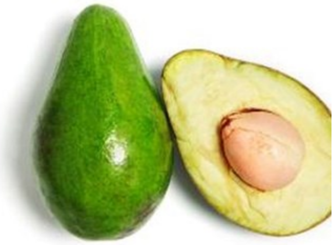
**Squash**	Calabacita/Zucchini: 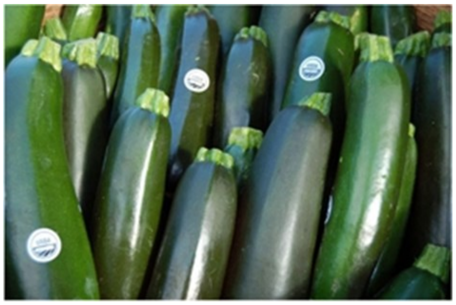	Chayote: 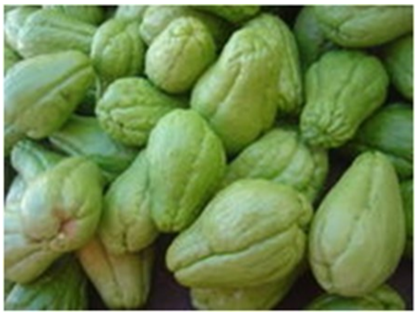
**Jicama**	Jicama: 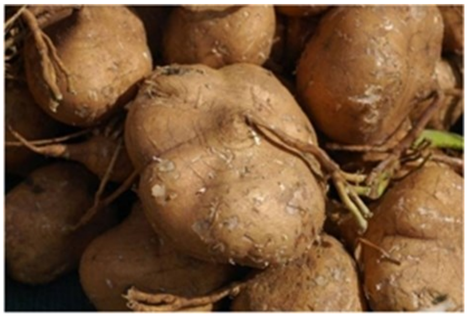	No Alternative
**Cactus/ Nopal**	Cactus Pad: 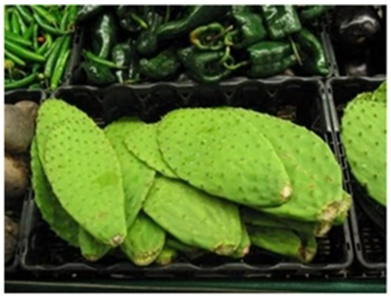	Sliced Cactus: 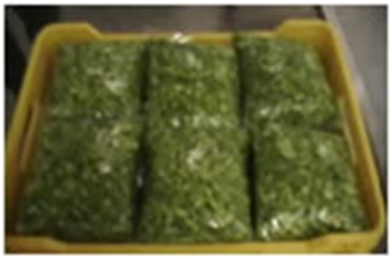

**Chiles**	Anaheim Chiles: 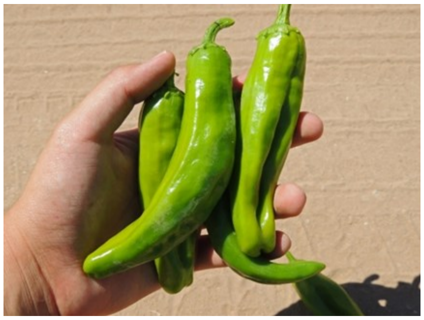	Poblano Chiles: 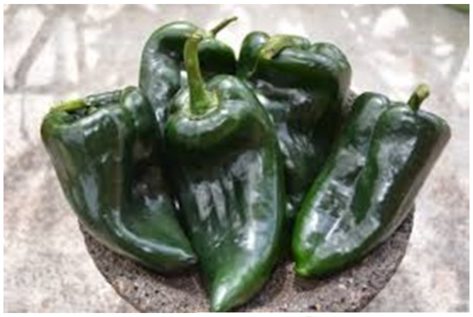 Pasilla Chiles: 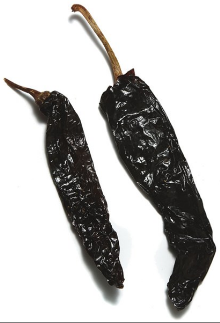
**Tomatillos**	Tomatillos: 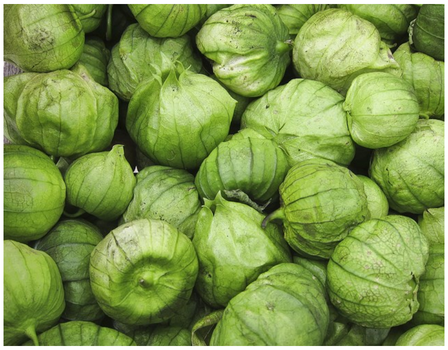	No Alternative

Tilapia

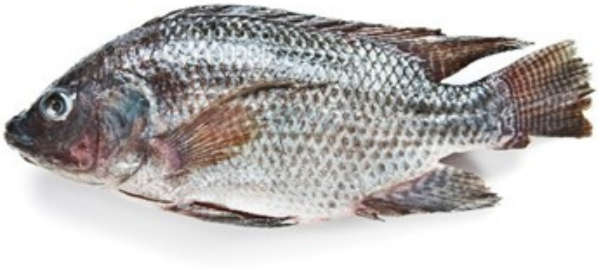

Salmon

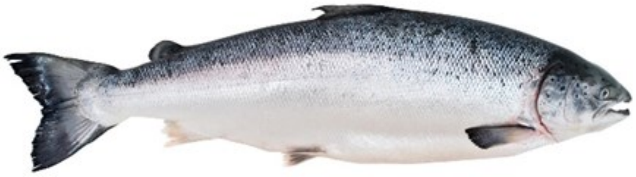

Cod

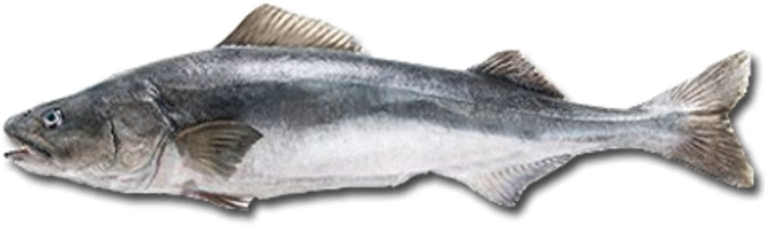

Mojarra

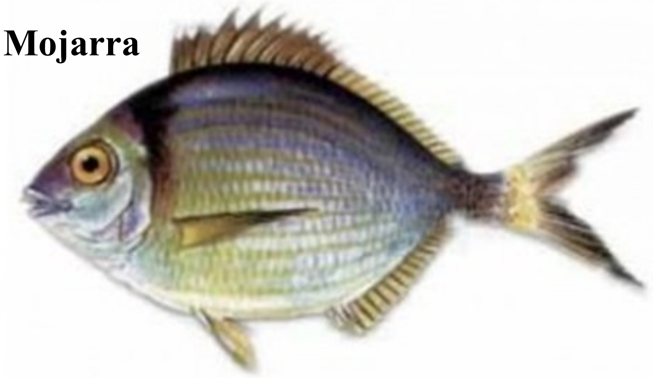

Supplement: Supplementary file 1 [file ijerph-19-01860-s001.zip › ijerph-1557439-supplementary.pdf]
